# Mouse pneumonia model by *Acinetobacter baumannii* multidrug resistant strains: Comparison between intranasal inoculation, intratracheal instillation and oropharyngeal aspiration techniques

**DOI:** 10.1371/journal.pone.0260627

**Published:** 2021-12-02

**Authors:** Gabriella Bergamini, Maria Elisa Perico, Stefano Di Palma, Daniela Sabatini, Filippo Andreetta, Rossella Defazio, Antonio Felici, Livia Ferrari

**Affiliations:** 1 Translational Microbiology, Antibacterial Discovery, Aptuit (Verona) S.r.l., *an Evotec Company* DD&D Research Centre, Verona, Italy; 2 *In vitro* Pharmacology, Aptuit (Verona) S.r.l., *an Evotec Company* DD&D Research Centre, Verona, Italy; 3 Pathology, Preclinical Development, Aptuit (Verona) S.r.l., *an Evotec Company*, DD&D Research Centre, Verona, Italy; 4 *In vitro* Pharmacology, Microbiology Discovery, Aptuit (Verona) S.r.l., *an Evotec Company*, DD&D Research Centre, Verona, Italy; Rutgers Biomedical and Health Sciences, UNITED STATES

## Abstract

Infectious pneumonia induced by multidrug resistant (MDR) *Acinetobacter baumannii* strains is among the most common and deadly forms of healthcare acquired infections. Over the years, different strategies have been put in place to increase host susceptibility to MDR *A*. *baumannii*, since only a self-limiting pneumonia with no or limited local bacterial replication was frequently obtained in mouse models. Direct instillation into the trachea or intranasal inoculation of the bacterial suspension are the techniques used to induce the infection in most of the preclinical models of pneumonia developed to date. More recently, the oropharyngeal aspiration procedure has been widely described in the literature for a variety of purposes including pathogens administration. Aim of this study was to compare the oropharyngeal aspiration technique to the intranasal inoculation and intratracheal instillation in the ability of inducing a consistent lung infection with two MDR *A*. *baumannii* clinical isolates in immunocompromised mice. Moreover, pneumonia obtained by bacteria administration with two out of three techniques, intratracheal and oropharyngeal, was characterised in terms of histopathology of pulmonary lesions, biomarkers of inflammation level and leukocytes cells infiltration extent after mice treatment with either vehicle or the antibiotic tigecycline. The data generated clearly showed that both strains were not able to colonize the lungs when inoculated by intranasal route. By contrast, the bacterial load in lungs of mice intratracheally or oropharyngeally infected significantly increased during 26 hours of monitoring, thus highlighting the ability of these strains to generate the infection when directly instilled into the lower respiratory airways. Furthermore, the intragroup variability of mice was significantly reduced with respect to those intranasally administered. Tigecycline was efficacious in lung bacterial load and cytokines release reduction. Findings were supported by semi-quantitative histopathological evaluation of the pulmonary lesions and by inflammatory biomarkers analysis. To conclude, both intratracheal instillation and oropharyngeal aspiration techniques showed to be suitable methods for inducing a robust and consistent pneumonia infection in mice when difficult MDR *A*. *baumannii* clinical isolates were used. Noteworthy, oropharyngeal aspiration not requiring specific technical skills and dedicated equipment, was proven to be a safer, easier and faster technique in comparison to the intratracheal instillation.

## Introduction

*Acinetobacter baumannii* is a Gram-negative, obligate aerobe, coccobacillus and one of the most prevalent causes of nosocomial infections [1]. The ability of *A*. *baumannii* to acquire antibiotic resistance mechanisms has allowed this organism to persist in hospital environments and has facilitated the global emergence of MDR strains. Classified as an ESKAPE pathogen (*Enterococcus faecium*, *Staphylococcus aureus*, *Klebsiella pneumoniae*, *Acinetobacter baumannii*, *Pseudomonas aeruginosa*, and *Enterobacter* spp), carbapenem-resistant *A*. *baumannii* was considered by the World Health Organization the number one critical priority pathogen for which new therapeutics were urgently required [2, 3]. A large number of *A*. *baumannii* resistance mechanisms are known, including enzymatic degradation of drugs, target modifications, multidrug efflux pumps, and permeability defects [4]. The accumulation of several resistance mechanisms in *A*. *baumannii* has gradually decreased the number of antibiotic classes able to treat the infections in clinical practice.

*A*. *baumannii* causes a wide range of infections in both hospitals and communities, including skin and soft tissue, urinary tract infections, meningitis, bacteraemia and pneumonia, with the latter being the most frequently reported infection in both settings [5]. Hospital-acquired pneumonia occurs most typically in patients receiving mechanical ventilation in the intensive care unit (ICU) setting. It has been thought that ventilator-associated pneumonia (VAP) caused by *A*. *baumannii* is due to the colonization of the airway via environmental exposure followed by the development of infection [6]. Nosocomial pneumonia happens as a result of aspiration. The presence of an endotracheal tube creates an ideal nidus for the environmental transmission of *Acinetobacter*, which avidly adheres to plastic and could establish the biofilm on the tube [7, 8]. Aspiration of droplets of *A*. *baumannii* directly into the alveoli of mechanically ventilated patients circumvents natural host barriers, allowing for establishment of infection in tissue.

Animal experimentation is still the most appropriate model to characterize the invading pathogen and the host response and to assess the efficacy of new antibacterial compounds during the pre-clinical phase of development. Indeed, different read-outs related to the infection such as mortality, tissue bacterial load, cytokine levels in bronchoalveolar lavage fluid, inflammatory cell infiltration into the lung and histological score can be measured.

*A*. *baumannii* strains as ATCC 17978 and 19606 have been used in the past to study lung infection pathogenesis *in vivo*, however, they do not represent the actual population of clinical strains isolated in the hospital. When compared to international clones Type I and II, both *A*. *baumannii* ATCC 17978 and 19606 showed a reduced virulence and elicited different immune responses [9–11].

Furthermore, many laboratory strains and clinical isolates of *A*. *baumannii* did not infect immunocompetent mice or, in case, induced only a self-limiting pneumonia with no or very limited local bacterial replication and systemic dissemination, even when a large inoculum was used [12–14]. To overcome these shortcomings, immunocompromised mice have been used to increase both host susceptibility and bacterial virulence. Despite that, the selection of *A*. *baumannii* strain and the technique used for bacteria delivering to the animals remains a critical step to obtain a sustainable and reliable pneumonia model.

Intranasal (IN) inoculation or intratracheal (IT) instillation of the bacterial suspension are procedures reported in almost all models developed to date.

In particular, IN inoculation, where the bacterial challenge is pipetted over the nares of anesthetized mice, has gained popularity because it is an easy technique suitable for repeated administrations [15, 16]. However, the disadvantage of this method is that the distribution between the upper and lower respiratory tracts is heavily influenced by the instilled volume and the degree of anaesthesia. Moreover, a portion of the intranasally delivered bacterial challenge could stay out of the lung, causing off-target complications and symptoms like sinusitis and physical trauma, which are inconsistent with the disease progression in human [17].

On the contrary, the intratracheal (IT) instillation allows the administration of a precise amount of bacteria locally into the lung. Many protocols describing this procedure include blind intubation of the trachea through the oral cavity or surgical exposure of the trachea to directly access the lung. Herein, a consistent, non-invasive and non-surgical method for IT instillation is reported. The opening of the trachea is visualized using a laryngoscope and a delivery tube is then inserted directly into the trachea of anesthetized animals to instill the inoculum dispersed by compressed air. High technical expertise and specific equipment are requested to perform the overall procedure and this could represent an important limitation [18, 19].

This is the reason why alternative techniques have been considered. The oropharyngeal (OP) aspiration technique may be used to simulate the natural route and pathogenesis of bacterial lung infection by closely mimicking pneumonia observed in human [20–22]. Thanks to the natural reflexive aspiration, this technique ensures that the entire challenge reaches specifically the lungs. A lung infection characterized by a uniform pulmonary distribution of bacteria and reduced intragroup variability can be obtained. Unlikely to IT instillation technique, the OP procedure is easier and safer since just a pipette is used to deliver the bacterial inoculum to the animal by decreasing the risk of suffocation [23].

Here, we describe a murine model of acute MDR *A*. *baumannii* pneumonia in immunocompromised mice where the ability in inducing a sustained lung infection of two clinical strains administered by means of the three previously described methods, IN, IT and OP has been evaluated. Then, the model induced by one out of the two MDR strains administered either intratracheally or oropharingeally has been characterised not only in terms of infection development in the lungs of animals, untreated and treated with vehicle (VEH) or tigecycline (TGC), but also by examining the lung inflammation through histopathological analysis and cytokines and leukocytes cells infiltration determination.

## Materials and methods

### Bacterial strains

The bacterial strains were from Aptuit (Verona) S.r.l., *an Evotec Company*, Bacterial Culture Collection.

Two virulent MDR *A*. *baumannii* clinical strains, namely *A*. *baumannii* ACC001 and *A*. *baumannii* ACC002, with different susceptibility to key antibiotics (Table 1A), isolated from sputum of two different hospitalized patients, were selected. Whole genome sequencing (WGS) was also carried out to identify the genetic elements characterising the resistome of the strains (Table 1B).

**Table 1:** Susceptibility (A) and resistance (B) profile of *A*. *baumannii* strains tested.

**A**								
	**MIC (μg/mL)**							
**Bacterial strain**	**CIP**	**CST**	**ERY**	**MEM**	**RIF**	**SUL**	**TGC**	**TOB**
*A*. *baumannii* ACC001	>64	0.25	>64	32	4	16	2	>64
*A*. *baumannii* ACC002	64	0.5	32	32	8	32	0.5	16
**B**								
**Bacterial Strain**	**Molecular Resistance Profile**							
*A*. *baumannii* ACC001	aph(3’’)-Ib, aph(6)-Id, tet(B), ant(3’’)-IIa, OXA-66, aac(6’)-Ib’, msr(E), mphE, catB8, aadA1, qacEdelta1, sul1, armA, ADC-30, OXA-23, aph(3’)-Ia							
*A*. *baumannii* ACC002	ADC-type, ant(3’’)-IIa, OXA-68, tet(B), aph(6)-Id, aph(3’’)-Ib, OXA-366, aac(3)-IIa, aac(6’)-Ian, TEM-1, sul2							

### Preparation of antibiotic stock solutions

A fresh stock solution of ciprofloxacin (CIP, Sigma Aldrich, Italy), colistin (CST, Sigma Aldrich, Italy), erythromycin (ERY, Sigma Aldrich, Italy) meropenem (MEM, Sigma Aldrich, Italy), rifampicin (RIF, Sigma Aldrich, Italy), sulbactam (SUL, Sigma Aldrich, Italy), TGC (Sigma Aldrich, Italy) and tobramycin (TOB, Sigma Aldrich, Italy) was prepared daily for the Minimum Inhibitory Concentration (MIC) assay.

For *in vitro* testing, antibiotic stock solution was prepared by dissolving the drugs in the appropriate solvent as described in Clinical and Laboratory Standards Institute (CLSI M100, 30^th^ Ed). The working solution of antibiotic was prepared at a concentration 100 fold higher that the desired final drug concentration.

For *in vivo* studies, TGC hydrate solution at concentration of 12.5 mg/mL was freshly prepared in sterile physiological saline (0.9% NaCl) and stored at 4 °C prior to use.

### Susceptibility testing

MICs of both clinical isolates to each drug were tested using the broth microdilution method, as recommended by the Clinical and Laboratory Standards Institute (CLSI, M07, 11^th^ Ed). A bacterial suspension (0.5 McFarland) was prepared in Mueller-Hinton (MH) broth, then diluted (ten-fold, twice) and aliquoted into 96-well plates. *Pseudomonas aeruginosa* ATCC^®^27853 was used as the quality control strains. For TGC, stock solution in sterile water was freshly prepared on the day of use.

### Inoculum preparation for lung infection

The day before infection, *A*. *baumannii* ACC001 and ACC002 cells from frozen stock, were grown on Tryptic Soy Agar plates (TSA, Thermo Fisher Scientific, Italy) plate at 37 °C overnight. On the day of infection the inoculum was prepared as follows: few colonies from the overnight agar plate were suspended in 5 mL of sterile Luria Broth medium (LB, Sigma Aldrich, Italy) and the turbidity of bacterial suspensions were adjusted to match the 0.5 McFarland standard (~10^8^ CFU/mL). Bacterial suspensions were opportunely diluted to obtain the concentration required for inducing the lung infection (*A*. *baumannii* ACC001 7.22 Log10 CFU/mL and ACC002 7.39 Log10 CFU/mL).

### Animals

All experiments involving animals were carried out in accordance with both the European directive 2010/63/UE governing animal welfare and protection, which is acknowledged by the Italian Legislative Decree no 26/2014 and the company policy on the care and use of laboratory animals. All the studies were revised by the Animal Welfare Body and approved by Italian Ministry of Health (authorization n. 51/2014-PR).

Male CD-1 mice (Charles River Laboratories, Italy), 6 weeks old, were allowed 5 days for acclimation after arrival. The mice were maintained on a 12-hour light cycle with *ad libitum* access to rodent feed (Altromin R, A. Rieper SpA, Italy) and filtered tap water. Animals were monitored during the entire period of the studies and clinical signs were recorded (S1 Table).

### Animal models of pneumonia

#### Induction of neutropenia

Cyclophosphamide Monohydrate (CPM, Sigma Aldrich, Italy) was dissolved in distilled water for injection to a final concentration of 1%. All mice were administered with a total dose of 250 mg/kg by two intraperitoneal injections scheduled at day -4 (150 mg/kg single dose) and day -1 (100 mg/kg single dose) [24]. Thereafter mice were randomly assigned to groups of infection and treatment.

#### Intranasal inoculation infection

Mice (n = 20) were transiently anaesthetized using an isoflurane vaporizer chamber set at 3.5% isoflurane mixed with oxygen and bacterial suspensions (50 μL) of *A*. *baumannii* ACC001 or ACC002 were gradually released into the nostrils with the help of a micropipette. The rate of release was adjusted so as to allow the mouse to inhale the inoculum without forming bubbles. To facilitate consistent inoculations, mice were held vertically during inoculation and for another couple of minutes till breathing gradually returned to normal.

#### Intratracheal instillation infection

Mice (n = 20) were anaesthetized with isoflurane and then suspended by their upper incisors from a nylon string on an angled plexiglass platform. Once the tracheal opening was visualized using the laryngoscope, the delivery tube was inserted gently into the trachea of the mouse. Bacterial suspensions (50 μL) of *A*. *baumannii* ACC001 or ACC002, loaded in a syringe fitted with the delivery tube, were instilled into the lungs by air compressed. Mice were hold upright for a few seconds to allow inoculum to be inhaled into the lungs.

#### Oropharyngeal aspiration infection

Mice (n = 20) were anaesthetized with isoflurane and then suspended by their upper incisors from a nylon string on an angled plexiglass platform. Curved forceps were used to gently open the mouth and hold the tongue down to the lower jaw to prevent swallowing. Once the tracheal opening was visualized using the laryngoscope, bacterial suspensions (50 μL) of *A*. *baumannii* ACC001 or ACC002 were carefully administered to the back of the mouth using a micropipette. Mice, obligate nasal breathers, were encouraged to open epiglottis for the aspiration of the instilled fluid.

#### Bacterial load determination

Animals (5 mice/group or 5 mice/strain/technique) were sacrificed by overdose of inhaled 5% isoflurane at 2 and 26 hours after the infection. Lungs and trachea were aseptically removed. The lungs were placed in pre-weighted tubes containing 4 mL of sterile physiologic saline and then homogenized with Cryolys^®^ Evolution Precellys system (Bertin Instruments, France). The tracheas were placed into 2 mL of sterile saline and homogenized with ULTRA-TURRAX^®^ homogenizer system (IKA, Germany). Tenfold dilutions of homogenates were plated on TSA plates and incubated overnight at 37°C. Results of cultures were expressed as Log10 of CFU/lungs and CFU/trachea. The lower limit of detection was 2.2 Log10 CFU/tissue. The mean counts ± the standard deviation of the Log10 CFU/tissue were obtained at 2 and 26 hours after induction of pneumonia, and the difference between the two timepoints was calculated (ΔLogs10 = mean _26 hours_ − mean _2 hours_).

Moreover blood (200 μl at least/ animal) was terminally collected by cardiac puncture and put into K_3_EDTA tubes at 2 and 26 hours after the infection. Both blood samples undiluted obtained at 2 hours after the infection and serially tenfold diluted in sterile saline collected after 26 hours of the infection, were plated on TSA plates and incubated overnight at 37°C for CFU/mL determination.

#### Antibiotic treatment

Mice (n = 70) were anaesthetized and intratracheally (n = 35) or oropharyngeally (n = 35) inoculated with 50 μL of *A*. *baumannii* ACC001 bacterial suspension (2 x 10^7^ CFU/mL). At 2 hours post infection, animals were subcutaneously treated with VEH (sterile saline) or TGC hydrate at 60 mg/kg every 12 hours (BID). Five mice/group were sacrificed at start and end of therapy (2 and 26 hours post infection, respectively). Lungs were aseptically removed and homogenized as described above. Dilutions of homogenates were plated on TSA plates and incubated overnight for CFU/lungs determination. The mean counts ± the standard deviation of the Log10 CFU/lungs were obtained for either VEH or TGC treated group (i.e. 26 hours after infection) and start of therapy group (i.e. 2 hours after infection), and the difference between the two groups was calculated (ΔLogs10 = mean _treated group_- mean _start of therapy group_). Bronchoalveolar lavage fluid (BALF) and lungs were collected from additional five mice/group at the end of therapy for leukocytes and cytokines determination and histopathology analysis respectively.

### Bronchoalveolar lavage fluid (BALF) collection and analysis

Lungs were washed three times with 0.4 mL of Dulbecco’s Phosphate Buffered Saline 0.01 M (DPBS, Sigma Aldrich, Italy) solution. BALF was kept on ice and then centrifuged at 400 x *g* at 4°C for 5 minutes. Cell pellets were suspended in 0.5 mL of DPBS and total cells counted by DASIT Sysmex XT-2000iV haemocytometer. An aliquot of BAL supernatant (~ 250 μl) was stored at -80°C for measurement of inflammation biomarkers level.

### Measurement of cytokine in mouse BALF

BALF cytokines—IFN-γ, IL-10, IL-12p70, IL-1β, IL-2, IL-4, IL-5, IL-6, KC/GRO, and TNF-α—were quantified by the MSD (Meso Scale Discovery) V-PLEX Proinflammatory Panel 1 (mouse) Kit (Cat. K15048D-1), according to the manufacturer’s protocol and measured with the MESO SECTOR^®^ Imager 6000.

### Histological examination

Lungs were removed, fixed in 10% neutral buffered formalin, embedded in paraffin blocks; thereafter 3.5 μm-thick sections including all pulmonary lobes were obtained and subsequently stained with Haematoxylin and Eosin (H&E). One section per animal (containing all lung lobes) was examined using an inverted bright field microscope at a magnification of × 600; relevant images were acquired by connecting the microscope to a camera. Several parameters (extension of inflammation, percentage of neutrophils and macrophages, distribution of inflammation, presence of haemorrhages, oedema, intracellular and extracellular bacteria, affected portions and numbers of affected lung lobes) were evaluated and graded independently by two board-certified veterinary pathologists on the same set of slides/sections (technical repeats), following examination of all the pulmonary tissue present on each slide/section. Re-evaluation of the sections with different grades assigned by the two pathologists was performed, reaching a final agreement for these cases.

### Statistical analysis

Data were shown as means and standard deviations (S.D.). Statistical analysis of CFU data was performed by a Two Way ANOVA followed by Tukey’s Multiple Comparison Test.

For both BALF leukocytes and cytokines quantification data were shown as means and standard deviations (S.D.) (n = 5): statistical analysis was performed by a Student’s T test (Graphpad Prism 8.1.1, Graphpad Software Inc., San Diego, USA). A level of p < 0.05 was considered significant.

## Results

### Timecourse of pneumonia induced by two MDR *A*. *baumannii* strains in mice IN, IT or OP infected

**Fig 1:**
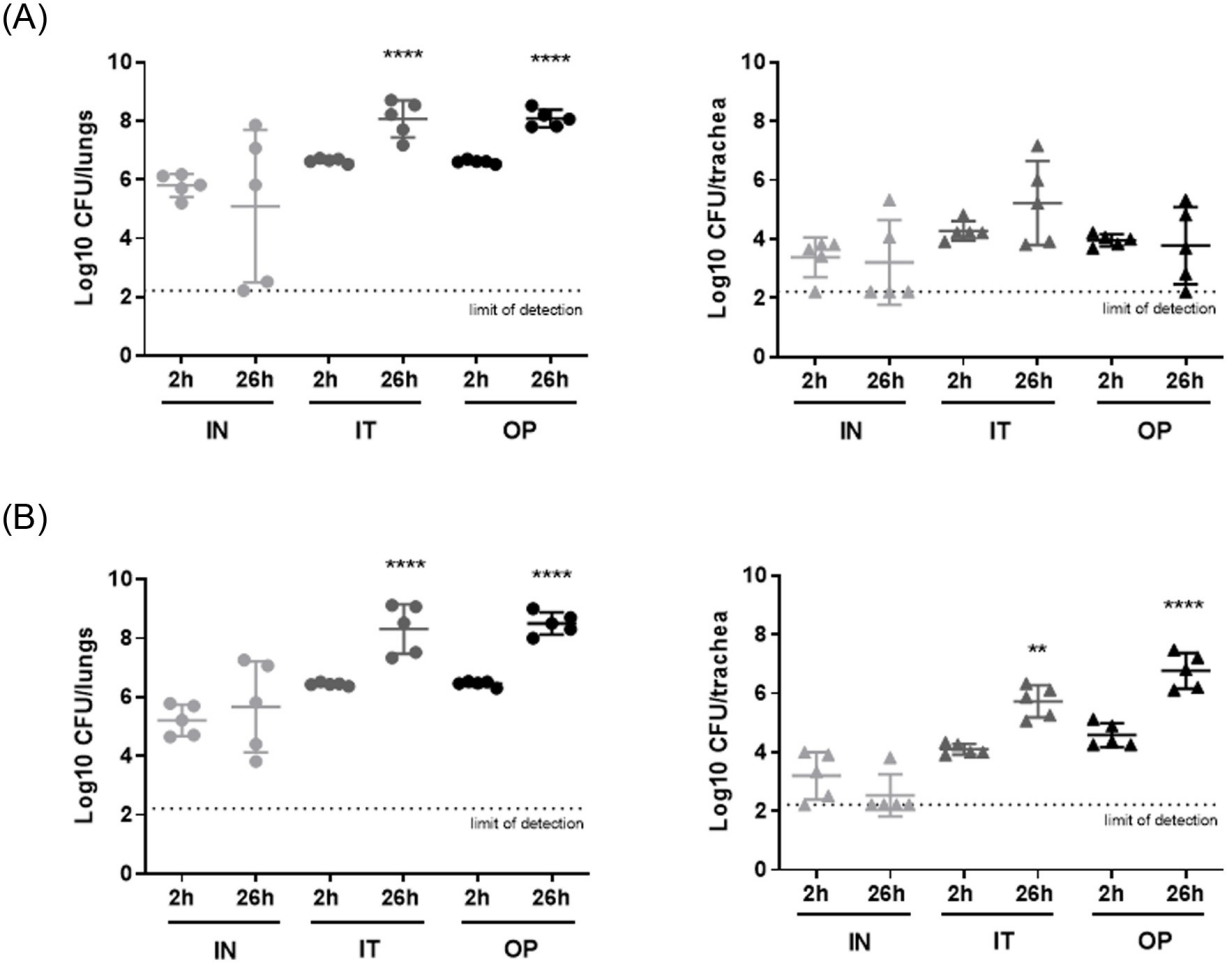
Bacterial load in lungs and trachea of immunocompromised mice infected with *A*. *baumanii* ACC001 (A) and ACC002 (B). Bacterial counts in lungs and trachea of animals infected with MDR *A*. *baumannii* strains ACC001 (challenge 5.92 Log10 CFU/mouse) and ACC002 (challenge 6.10 Log10 CFU/mouse) with intranasal (IN), intratracheal (IT) or oropharyngeal (OP) techniques. Data are expressed as scatterplot distribution (included mean). Statistical analysis was performed by a One Way ANOVA followed by Tukey’s Multiple Comparison Test, ****p<0.0001, **p<0.01 (26 hours *vs* 2 hours post infection for each infection technique).

The bacterial load in lungs and trachea induced by similar challenges of two MDR *A*. *baumannii* clinical strains ACC001 and ACC002 (5.92 and 6.10 Log10 CFU/mouse, respectively) IN, IT or OP administered to immunocompromised mice was assessed over 26 hours of infection (Fig 1A and 1B, Tables 2 and 3).

**Table 2:** Bacterial load in tissues of *A*. *baumannii* ACC001 infected mice.

	Log10 CFU/lungs			Log10 CFU/trachea	
Infection	Timepoint (hours post infection)	Bacterial load (average ± SD)	ΔLog10 CFU/lungs *vs* 2 hours post infection	Bacterial load (average ± SD)	Δ Log10 CFU/trachea *vs* 2 hours post infection
**IN**	2	5.80 ± 0.39	-0.71	3.39 ± 0.67	-0.18
	26	5.10 ± 2.60		3.21 ± 1.43	
**IT**	2	6.64 ± 0.08	1.43	4.28 ± 0.33	0.95
	26	8.07 ± 0.63		5.23 ± 1.42	
**OP**	2	6.61 ± 0.06	1.47	3.97 ± 0.20	-0.19
	26	8.08 ± 0.30		3.78 ± 1.31	

**Table 3:** Bacterial load in tissues of *A*. *baumannii* ACC002 infected mice.

	Log10 CFU/lungs			Log10 CFU/trachea	
Infection	Timepoint (hours post infection)	Bacterial load (average ± SD)	Δ Log10 CFU/lungs *vs* 2 hours post infection	Bacterial load (average ± SD)	Δ Log10 CFU/trachea *vs* 2 hours post infection
**IN**	2	5.21 ± 0.53	0.46	3.20 ± 0.81	-0.66
	26	5.67 ± 1.54		2.54 ± 0.72	
**IT**	2	6.44 ± 0.05	1.87	4.10 ± 0.18	1.63
	26	8.31 ± 0.84		5.73 ± 0.55	
**OP**	2	6.46 ± 0.09	2.04	4.58 ± 0.40	2.19
	26	8.50 ± 0.38		6.77 ± 0.60	

Animals intranasally challenged did not show a significant difference in terms of lung bacterial counts detected after 26 hours in comparison to 2 hours of infection (0.71 Log10 CFU/lungs of reduction and 0.46 Log10 CFU/lungs of increase, with ACC001 and ACC002, respectively). Obtained read out was coupled with a huge intra-group variability confirming that both clinical strains were not able to induce a sustained infection. By contrast, the lung infection was correctly established when both *A*. *baumannii* isolates were administered through IT instillation or OP aspiration. A comparable, significant, and homogeneous bacterial increase of more than 1.40 and 1.80 Log10 CFU/lungs on average was detected in mice infected with ACC001 and ACC002, respectively, after 26 hours of infection independently on the technique used. Similarly, trachea showed to be colonized in all tested conditions even if the bacterial load recorded was lower in comparison to those detected in the lungs. Then, while static or reduced bacterial counts were detected in the trachea of mice IN infected with both strains and IT or OP challenged with *A*. *baumannii* ACC001, a significant increase was observed in animals IT or OP infected with *A*. *baumanni* ACC002 after 26 hours (1.63 Log10 CFU/lungs and 2.19 Log10 CFU/lungs, respectively). The infection induced by IN, IT and OP administered strains was localised in the airways as the bacterial counts in blood were below limit of detection (data not shown).

As far as clinical monitoring is concerned, all mice infected with *A*. *baumannii* ACC001 and ACC002 by IN inoculation showed a body weight loss characterized by a high intra-group variability (6.67 ± 6.38% and 1.72 ± 6.20% on average ± SD, respectively) and just a mild piloerection during 26 hours of infection.

On the other hand, mice challenged by IT and OP technique showed a higher and homogeneous body weight loss with respect to IN administered animals when infected with *A*. *baumannii* ACC001 (15.96 ± 0.83% and 15.01 ± 1.40% on average ± SD, respectively) or *A*. *baumannii* ACC002 (14.35 ± 3.36% and 15.63 ± 1.43% on average ± SD, respectively) and the clinical score increased since moderate piloerection, slightly reduced motor activity and tachypnea were detected over 26 hours of infection (Table 4, S2 Table).

**Table 4:** Body weight and clinical score.

	BW loss (end *vs* start of infection) at 26 hours (% average ± SD)		Clinical score median at 26 hours	
Infection	*A*. *baumannii* ACC001	*A*. *baumannii* ACC002	*A*. *baumannii* ACC001	*A*. *baumannii* ACC002
**IN**	6.67 ± 6.38	1.72 ± 6.20	4	1
**IT**	15.96 ± 0.83	14.35 ± 3.36	8	8
**OP**	15.01 ± 1.40	15.63 ± 1.43	7	8

### Antibiotic efficacy assessment in acute pneumonia model induced by IT and OP administration of *A*. *baumannii* ACC001

**Fig 2:**
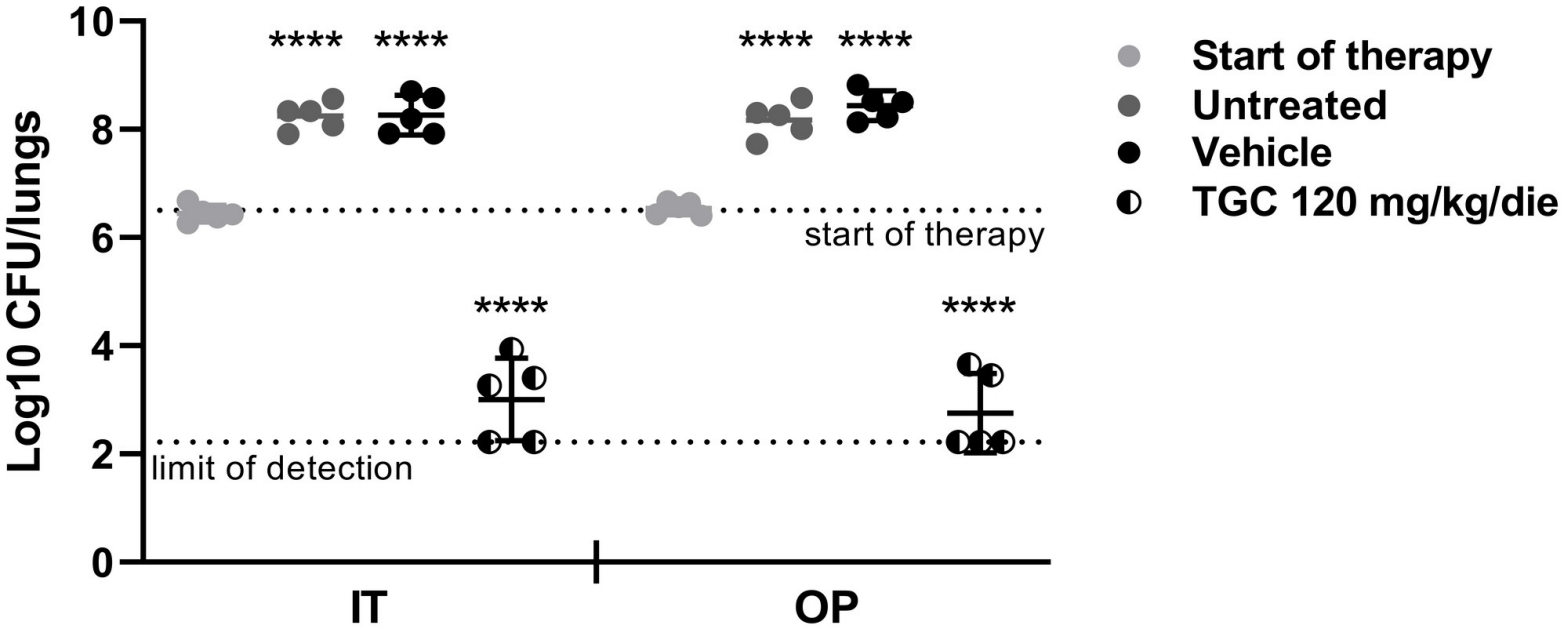
Bacterial load in lung of immunocompromised mice infected with *A*. *baumanii* ACC001 during therapy with TGC. Bacterial counts in lungs of animals intratracheally (IT) and oropharyngeally (OP) infected with MDR *A*. *baumannii* ACC001 (challenge 5.77 Log10 CFU/mouse) untreated (T0) and treated with vehicle (VEH) or tigecycline (TGC) 120 mg/kg/day (T24). Data expressed as scatterplot distribution (included mean). Statistical analysis was performed by a Two Way ANOVA followed by Tukey’s Multiple Comparison Test, ****p<0.0001 (VEH and TGC *vs* start of therapy).

Only one out of the two strains was progressed to the second phase of the current investigation since no difference in terms of susceptibility profile and timecourse of infection was highlighted. The bacterial counts in lungs of animals IT and OP infected with a suspension of *A*. *baumannii* ACC001 before and after treatment with VEH and TGC at 120 mg/kg/day are reported in Fig 2 and Table 5. A bacterial load of 6.44 Log10 CFU/lungs and 6.54 Log10 CFU/lungs on average after IT instillation or OP aspiration, respectively, was detected at 2 hours post infection corresponding to the start of therapy (T0). Both techniques induced a sustained pneumonia since bacterial counts increased by 1.90 Log10 CFU/lungs on average in infected VEH treated animals after 24 hours of treatment (T24) with respect to the start of therapy independently on the technique used. Similarly, TGC was efficacious in reducing bacterial load in lungs by more than 3.00 Log10 CFU/lungs after 24h of treatment.

**Table 5:** Bacterial load in lungs of *A*. *baumannii* ACC001 infected mice treated with vehicle and tigecycline.

Infection	Therapy	Timepoint (hours post start of treatment)	Log10 CFU/lungs (average ± SD)	Δ Log10 CFU/lungs vs start of therapy
**IT**	/	0	6.44 ± 0.16	/
	Untreated	24	8.24 ± 0.26	1.80
	VEH	24	8.27 ± 0.36	1.83
	TGC 120 mg/kg/day	24	3.01 ± 0.76	-3.43
**OP**	/	0	6.54 ± 0.12	/
	Untreated	24	8.18± 0.32	1.64
	VEH	24	8.44 ± 0.28	1.90
	TGC 120 mg/kg/day	24	2.75± 0.73	-3.79

IT and OP infected VEH treated mice showed a moderate body weight loss (13.8 ± 0.8 and 14.1 ± 0.7%, respectively) and a clinical status characterized by marked reduced motor activity and slight decreased respiratory rate, while, a mild body weight reduction (less than 8%) and only a mild piloerection were observed in animals treated with TGC (Table 6, S3 Table).

**Table 6:** Body weight and clinical score.

Infection	Therapy	BW loss (end *vs* start of treatment) at 24 hours (% average ± SD)	Clinical score median at 24 hours after start of therapy
**IT**	Untreated	-12.0 ± 1.4	8
	VEH	-13.8 ± 0.7	8
	TGC 120 mg/kg/day	-4.9± 2.0	1
**OP**	Untreated	-13.1 ± 2.3	8
	VEH	-14.1 ± 2.1	8
	TGC 120 mg/kg/day	-7.1 ± 2.8	2

### Host immune response to *A*. *baumannii* ACC001

BALF leukocytes, collected from mice infected via IT and OP, were analysed with the objective to assess the inflammatory cells recruitment in the lung. White Blood Cells (WBCs) and their relative sub-populations (Lymphocytes, Neutrophils, Monocytes, Eosinophils and Basophils) were counted by haemocytomer (Table 7). The high number of lung WBC infiltration in all infected mice confirmed a comparable level of inflammatory response in all groups of treatment. Indeed, total WBC count means were, (0.375±0.30) x 10^6^ in IT-infected VEH-treated mice, (0.387±0.30) x 10^6^ in IT infected TGC-treated mice, (0.363±0.15) x 10^6^ in OP infected VEH-treated mice and (0.253±0.13) x 10^6^ in OP infected TGC-treated mice without any significant difference among groups (p>0.05). WBC sub-population counts indicated that the inflammatory response was mainly sustained by neutrophils and monocytes infiltration in the lung.

**Table 7:** BALF leukocytes total counts.

	BALF Leukocyte total count (mean x 10^6 ± SD)			
	IT		OP	
	VEH	TGC 120 mg/kg/day	VEH	TGC 120 mg/kg/day
**LYMPHOCYTES**	0.050±0.095	0.010±0.010	0.018±0.021	0.006±0.004
**NEUTROPHILS**	0.080±0.063	0.155±0.135	0.143±0.084	0.092±0.050
**MONOCYTES**	0.209±0.163	0.196±0.144	0.177±0.077	0.136±0.061
**EOSINOPHILS**	0.008±0.004	0.010±0.008	0.006±0.004	0.004±0.007
**BASOPHILS**	0.028±0.025	0.016±0.016	0.019±0.010	0.015±0.009

**Fig 3:**
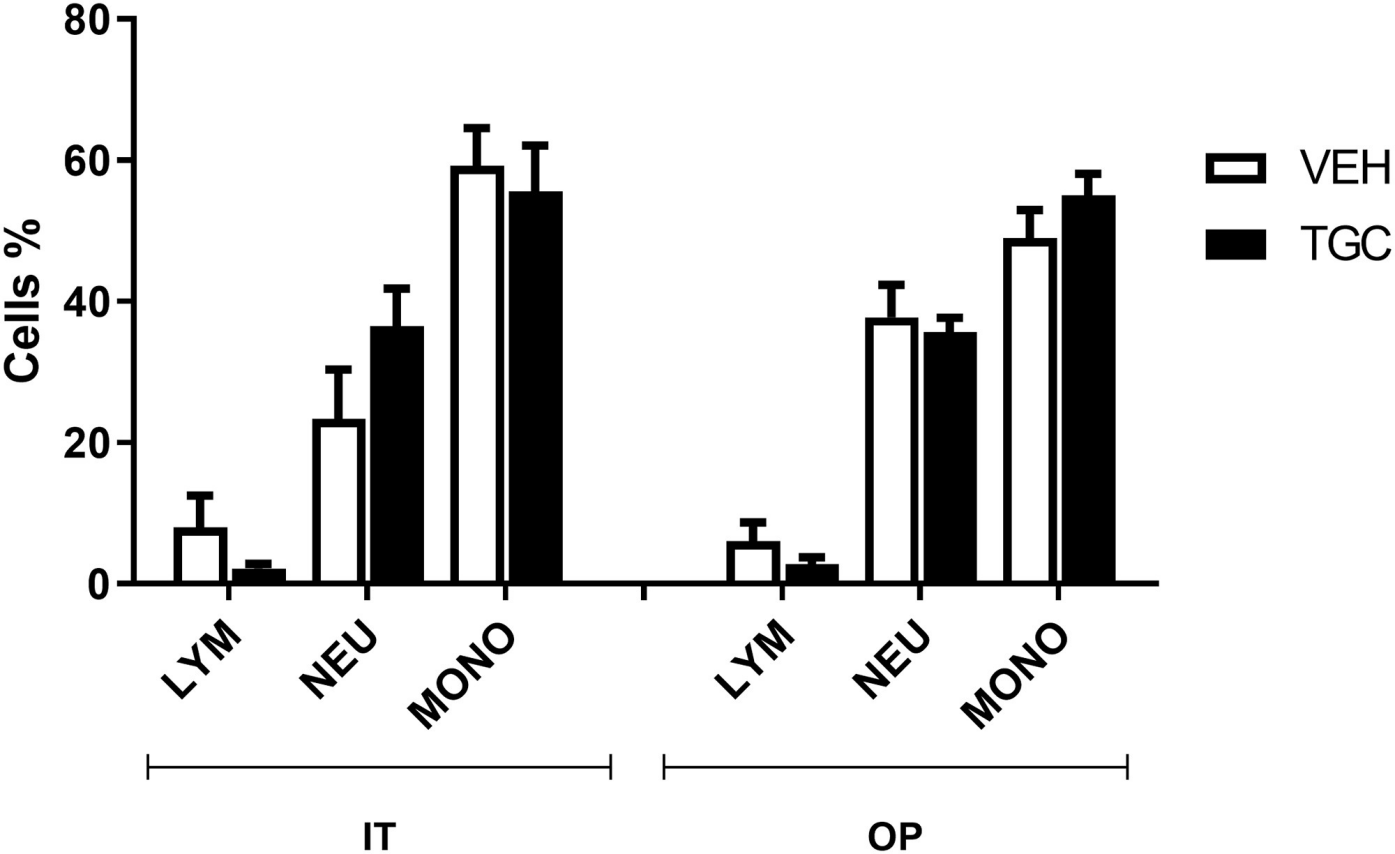
Lung infiltrating leukocytes in immunocompromised mice, upon IT or OP infection with *A*. *baumannii* ACC001. Lung BALF Leukocyte sub-population distribution, in intratracheally (IT) or oropharyngeally (OP) infected mice, treated with vehicle (VEH) and tigecycline (TGC) 120 mg/day. Data expressed as percentage (%) ±SD of total Leukocytes. Abbreviations. LYM: Lymphocytes; NEU: Neutrophils; MONO: Monocytes. Statistical analysis was performed by by two tailed Student’s T test considering significant p≤ 0.05.

The percentage of the more representative WBC sub-populations (Lymphocytes, Neutrophils and Monocytes) was found similar both in IT and OP challenged mice, thus indicating that there is not any significant difference (p>0.05) between the two techniques (Fig 3). Moreover, the treatment with TGC did not significantly affect the percentages of WBC subpopulations in comparison with VEH administered animals, both upon IT or OP infection, thus indicating the inflammatory response, induced by the infection, was not hampered by the antibiotic treatment.

Basophils and Eosinophils showed a comparable profile (Table 7).

### Measurement of cytokines in BALF of *A*. *baumannii* ACC001 infected mice

Cytokines were analysed in order to evaluate both the role played by the technique used to induce the infection (IT *vs* OP) on the establishment of the innate immune response in mice and the effect of treatment with TGC on the immune response induced by the infection.

**Fig 4:**
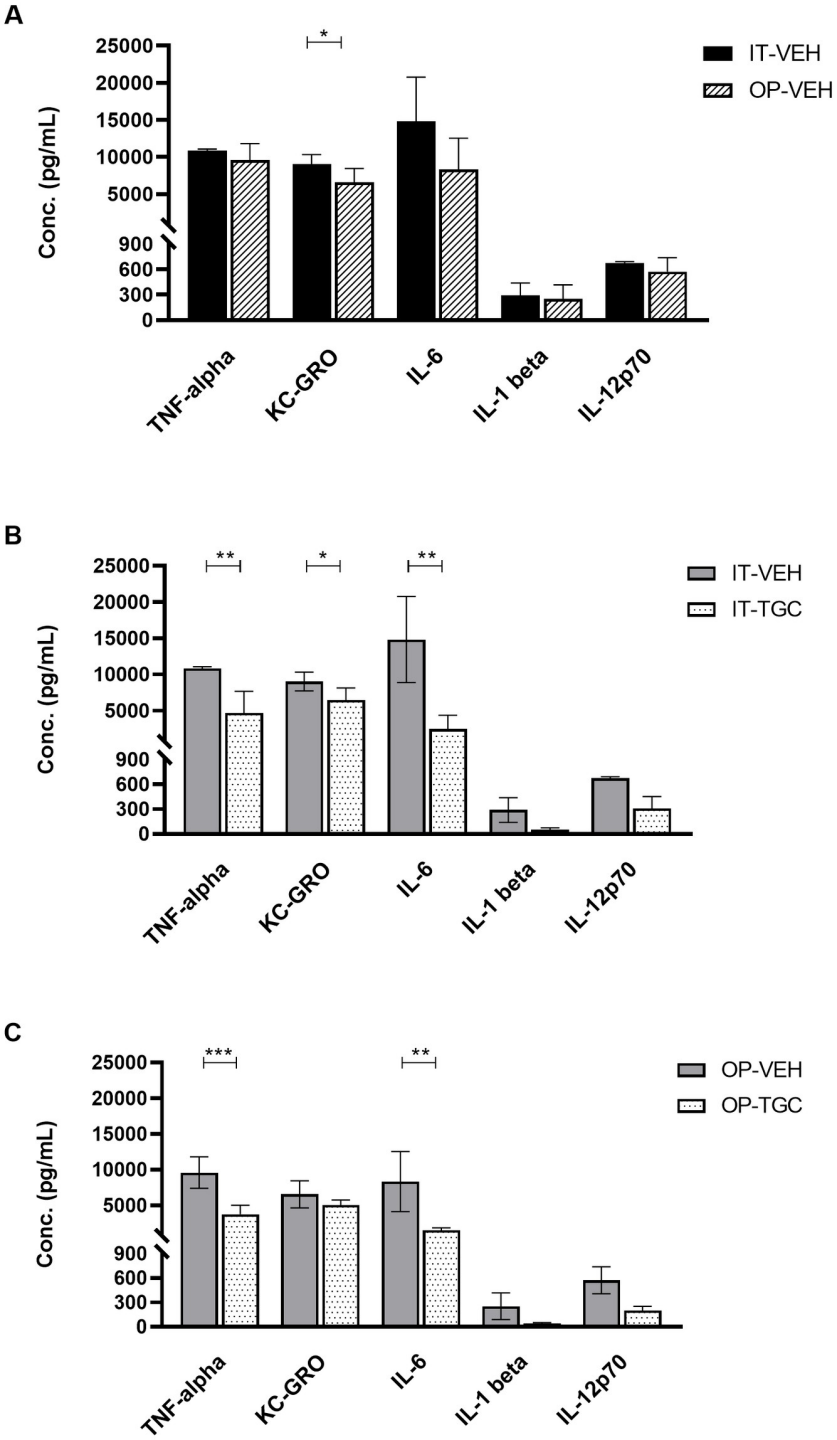
Cytokine quantification in BALF of *A*. *baumannii* ACC001 infected mice. Lung BALF cytokine quantification in vehicle (VEH) treated infected mice to assess the role played by the route of the infection intratracheal vs oropharyngeal (IT vs OP) (A) and to compare the effect of the tigecycline vs vehicle (TGC vs VEH) on the establishment of the innate immune response in intratracheally (IT) and oropharyngeally (OP) infected mice (B and C). Data expressed as mean ±SD. Statistical analysis was performed by two tailed Student’s T test considering significant p≤ 0.05.

VEH treated mice showed a very strong release of TNF-α, KC/GRO and IL-6, a moderate to high release of IL-1β and IL12/p70 (Fig 4A) but any release of IL-2, IL-4, IL-5, IL-10 or very low level of IFN-γ (S4 Table) independently on the technique used to challenge the animals. This cytokine pattern indicates the involvement of the innate immune response mainly sustained by Neutrophils and Monocytes, as confirmed by the BALF leukocytes count. Additionally, patterns of the cytokines released following IT or OP infection were comparable suggesting that the technique used did not alter the innate immune response to *A*. *baumannii* ACC001. Finally, the influence of TGC therapy on the immune response induced by the infection was evaluated. The data showed that treatment with TGC significantly reduced the amount of all the cytokines released in BALF of IT (Fig 4B) or OP infected mice (Fig 4C).

### Histopathological analysis

**Fig 5:**
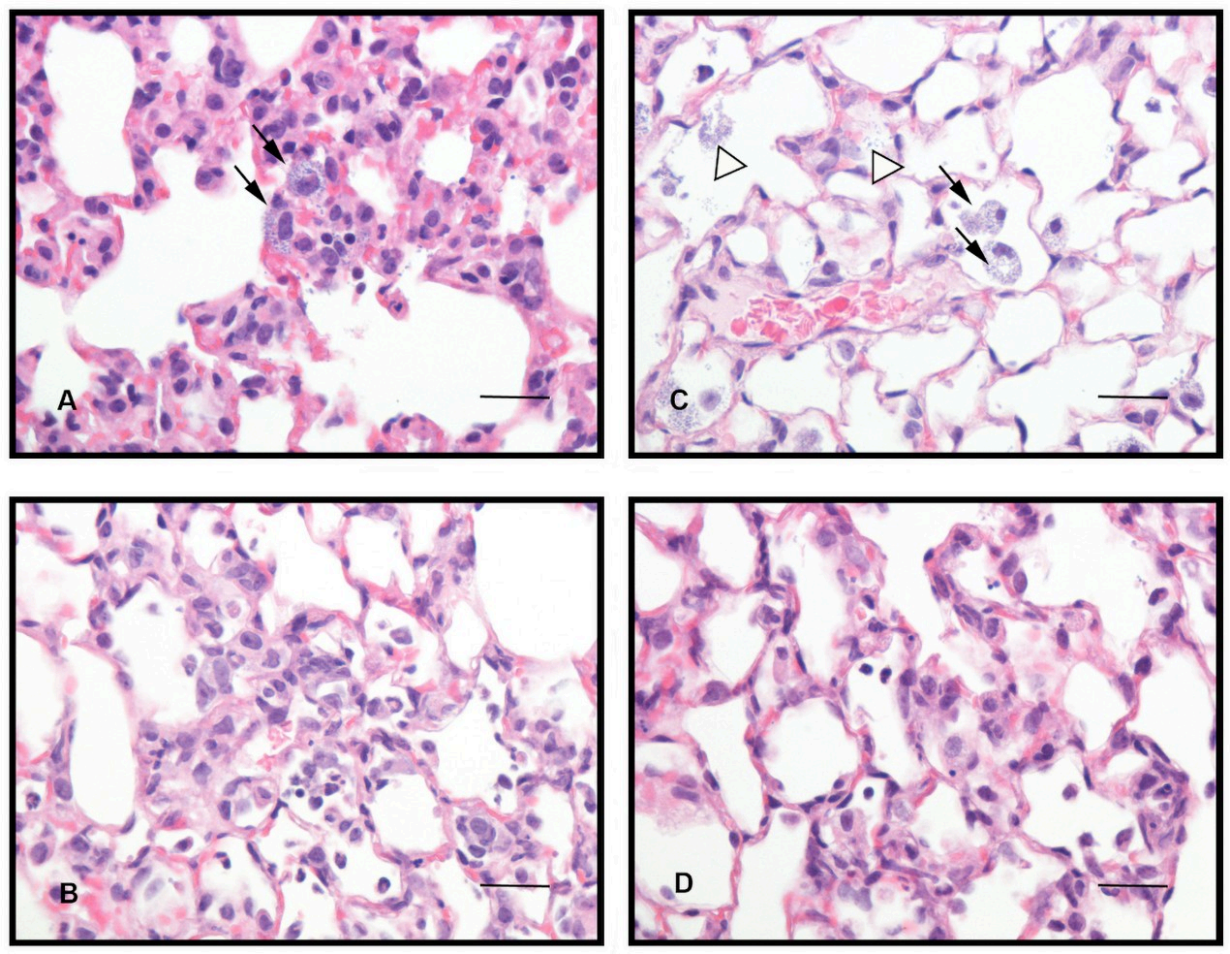
Sections of pulmonary parenchyma after IT and OP infection with *A*. *baumannii* ACC001 of VEH and TGC treated mice. Extracellular and intracellular bacteria were detected in untreated mice infected with both intratracheal (IT) (A) and oropharyngeal (OP) (C) techniques, with concurrent inflammation (hematoxylin and eosin staining, 600x magnification). Arrows indicate presence of bacteria in the cytoplasm of macrophages (black arrows) and in the extracellular space (white arrows). No bacteria were noted in mice treated with tigecycline (TGC) 120 mg/day and challenged by both intratracheal (IT) (B) and oropharyngeal (OP) (D) routes, with inflammatory changes similar in severity and distribution to untreated animals (hematoxylin and eosin staining, 600x magnification). All scale bars represent 30μm.

Mice infected with *A*. *baumannii* ACC001 by means of both IT and OP techniques and TGC and VEH treated (Fig 5A–5D) showed comparable pulmonary pathological findings. An inflammatory infiltrate mainly composed by macrophages (more than 50% of the infiltrate) with low number of neutrophils (up to 20% of the infiltrate) was seen, targeting almost exclusively the perivascular and alveolar spaces, and sparing bronchi, bronchioles and subpleural areas. Inflammatory changes were present in more than 5 fields at 200x in the majority of the examined pulmonary samples and frequently affected more than 2 of the 6 lobes of the lung (both hilar and central parenchyma). Intracellular (mainly in the cytoplasm of macrophages) and extracellular bacteria were only detected in VEH treated and not in TGC treated animals (Fig 5C and 5D).

## Discussion

*Acinetobacter baumannii* has emerged as one of the most successful Gram-negative bacteria causing both nosocomial and community-acquired infections globally. These infections are associated to prolonged hospitalization, tremendous healthcare costs and high mortality rate despite treatment [1]. Moreover, *A*. *baumannii* infections have become increasingly difficult to treat because of the rapid development of resistance against the most used antibiotics [2, 3]. Therefore, there is an urgent need to develop novel therapeutics and innovative intervention strategies to combat infections induced by this challenging pathogen. Animal models of *A*. *baumannii* infection play a critical role during the pre-clinical development phase of novel therapeutic approaches and compounds being instrumental to assess efficacy and safety before clinical trials. In most models developed to date, pneumonia infection is induced either through direct instillation into the trachea or by intranasal administration of a bacterial suspension. There is no evidence supporting the idea that one out of the two inoculation techniques (IT *vs*. IN) more accurately reproduces the human disease.

In this paper, we aimed to compare the use of OP aspiration to IN inoculation and IT instillation in the ability to induce a robust pneumonia model of infection with two MDR *A*. *baumannii* strains in immunocompromised mice.

The OP aspiration procedure has been widely described in the literature for a variety of purposes including administration of pathogens such as *Influenza A virus* and *Aspergillus fumigatus* [25, 26], toxins [27], bleomycin to induce lung fibrosis [28] and asthma [29, 30]. Previous studies, where OP was the technique used, demonstrated through a variety of methods that aspirated material preferentially deposited in the lungs and not in the mouth or stomach [28, 31].

Our results showed that IN inoculation of both *A*. *baumannii* ACC001 and ACC002 isolates was not a reliable technique; by contrast, a sustainable infection was obtained in lungs after both IT and OP bacterial challenge administration, where a similar bacterial growth was recorded after 26 hours of infection. (Fig 1A and 1B).

Early neutrophils recruitment to the lungs has been reported to be critical for *A*. *baumannii* clearance and depletion of neutrophils has been associated to high bacterial load, extrapulmonary dissemination and severe disease in the infected mice [32, 33].

Despite the pneumonia timecourse induced by *A*. *baumannii* ACC001 was evaluated in immunocompromised mice, where measured total WBC count resulted lower than those observed in immunocompetent animals [32, 34], obtained findings demonstrated that the inflammatory response in the lungs of mice was mainly sustained by neutrophils and monocytes infiltration after both IT and OP challenge. No further relevant difference was observed in terms of inflammatory cells migration (Fig 3).

The recruitment of neutrophils in the lungs during the infection is a multistep process coordinated by the production of chemoattractants and the expression of adhesion molecules by endothelial cells near the site of inflammation/infection. Since no significant difference of BAL levels of cytokines and chemokines such as TNF-α, IL–6, IL-1β and IL12/p70 (Fig 4A) was highlighted between mice infected by IT and OP technique, the innate immune response stimulated by *A*. *baumannii* ACC001 was not dependent on the technique used. The assumption that the response against *A*. *baumannii* was mainly due to the activation of innate immune system, was additionally clearly supported by the lack of the release of the cytokines (i.e. IL-2, IL-4, IL-5, IL10 and IFNγ) produced by the mediators of the specific immune response.

TGC’s ability to overcome resistance mechanisms to tetracyclines provides an opportunity for its use in nosocomial infections induced by MDR *Acinetobacter* spp [35]. *In vitro* data from several studies showed that TGC is active against resistant strains of *Acinetobacter* spp [36, 37]. In this study, TGC showed comparable efficacy in reducing the bacterial load in lungs of mice infected with a suspension of *A*. *baumannii* ACC001 IT or OP administered.

The high number of lung WBC infiltrated in TGC treated mice demonstrated that the administration of the antibiotic did not significantly affect the percentages of WBC subpopulations in comparison with VEH treated animals, when either IT or OP technique was used. On the contrary, treatment with TGC significantly reduced the amount of all the cytokines released in BALF of mice infected by IT or OP technique. This effect might be due to either an inhibition of *A*. *baumannii* growth [38] or to a modulation of the mice innate immune response [39, 40].

Regarding this last hypothesis, it has been previously reported that TGC showed rapid intracellular penetration into PMNs, due to the lack of many detoxification mechanisms [39]. Therefore, it is possible that TGC accumulated in PMNs may directly and/or indirectly alter the host-defence functions of leukocytes cells. Furthermore it has been described in literature that TGC affected the gene expression in LPS-activated PMNs by reducing the mRNA levels of five genes (TLR4, CD14, TNFα, IL6, and ITGAM) slightly and of TLR2 and IL8Rs significantly [40].

Histopathologic examination of the lungs of infected mice confirmed the pathogenicity of the clinical strain *A*. *baumannii* ACC001 and the marked inflammatory response characterized by a macrophage-rich inflammatory infiltrate and the lack of effect of TGC treatment.

To conclude, both IT instillation and OP aspiration techniques are consistent methods suitable for initiating robust pneumonia infections in mice with difficult MDR strains. Nevertheless, OP aspiration shows some important advantages when compared to the IT instillation since is a safer, easier and faster technique not requiring any specific technical skills and dedicated equipment. For this reason, our suggestion is to compare furtherly the two techniques in the ability of inducing pneumonia with additional pathogens of clinical interest to set up consistent, simple and really predictive experimental pneumonia models.

## Supporting information

S1 Table Clinical scoring system. The clinical scoring system adopted to assess the clinical signs observed in infected animals monitored.
(DOCX)

S2 Table Timecourse of lung infection: Body weight and clinical score values of mice. Individual and mean values of body weight and clinical score signs recorded during the timecourse of lung infection induced by MDR *A*. *baumannii* strains challenged by intranasal (IN), intratracheal (IT) or oropharyngeal (OP) techniques.
(DOC)

S3 Table Evaluation of TGC efficacy in acute pneumonia model: Body weight and clinical score values of mice. Individual and mean values of body weight and clinical score signs recorded during the study for the evaluation of tigecycline (TGC) efficacy in acute pneumonia model induced by intratracheal (IT) and oropharyngeal (OP) administration of *A*. *baumannii* ACC001.
(DOC)

S4 Table BALF cytokines quantification. Lung BALF cytokine quantification in mice infected mice by intratracheal (IT) and oropharyngeal (OP) techniques and vehicle (VEH) or tigecycline (TGC) treated. Data expressed as mean concentration (pg/mL) ±SD. Abbreviations. IFN-gamma: interferon-gamma; IL: interleukin; KC/GRO: keratinocyte chemoattractant/human growth regulated oncogene; TNF-alpha: tumor necrosis factor-alpha.
(DOC)
