# Supplementary material for: Mouse pneumonia model by Acinetobacter baumannii multidrug resistant strains: Comparison between intranasal inoculation, intratracheal instillation and oropharyngeal aspiration techniques
Source: PLoS One. 2021 Dec 2;16(12):e0260627. doi: 10.1371/journal.pone.0260627 (PMC8638993; doi:10.1371/journal.pone.0260627)
Supplement: S1 Table — The clinical scoring system adopted to assess the clinical signs observed in infected animals monitored. (DOCX) [file pone.0260627.s001.docx]

# **S1 Appendix. Clinical scoring system**

Animals are monitored regularly for signs of infection and scored according to value reported in the table below:

| **Variable** | **Score and description** |
| --- | --- |
| Body weight | 0- weight lost <5% |
|  | 1- weight lost 6-10% |
|  | 2- weight lost 11-15% |
|  | 3- weight lost 16-20% |
|  | 4- weight lost >20% |
| Appearance | 0- coat is smooth |
|  | 1- patches of hair piloerected |
|  | 2- majority of back is piloerected |
|  | 3- piloerection, mouse appears "puffy" |
|  | 4- piloerection, mouse appears emaciated |
|  | 5- skin lesions, such as lump, ulcer |
| Activity | 0- normal activity |
|  | 1- slightly reduced activity |
|  | 2- marked reduced activity |
|  | 3- severely impaired activity |
| Posture | 0- normal |
|  | 1- slightly hunched , moving freely |
|  | 2- hunched with activity |
|  | 3- hunched without activity |
|  | 4- ventral/lateral decubitus |
| Respiration rate | 0- normal, rapid mouse respiration |
|  | 1- slightly decreased respiration |
|  | 2- moderately reduced respiration |
|  | 3- severely reduced respiration |
|  | 4- asphyxia |
| Eyes | 0- open |
|  | 1- eyes not fully open, possibly with secretions |
|  | 2- eyes half closed or more, possibly with secretions |
|  | 3- eyes closed or milky |

Body weight (percentage of body weight loss over time) and clinical scores (total summary of signs) were expressed in terms of median and mean.

Mice were monitored daily and, based on the individual total scoring, the actions reported in the table below were taken to reduce the suffering and ensured the animal welfare.

| **Score** | **Actions** |
| --- | --- |
| >10 | Review frequency of monitoring |
| 11-15 | Check BT (<4°C implement HEP) - Consider supplementary care, e.g. extra fluids |
| >15 | Consult veterinarian |
| >20 | Implement humane endpoint (HEP) |
