# Supplementary material for: Mouse pneumonia model by Acinetobacter baumannii multidrug resistant strains: Comparison between intranasal inoculation, intratracheal instillation and oropharyngeal aspiration techniques
Source: PLoS One. 2021 Dec 2;16(12):e0260627. doi: 10.1371/journal.pone.0260627 (PMC8638993; doi:10.1371/journal.pone.0260627)
Supplement: S2 Table — Individual and mean values of body weight and clinical score signs recorded during the timecourse of lung infection induced by MDR A. baumannii strains challenged by intranasal (IN), intratracheal (IT) or oropharyngeal (OP) techniques. (DOC) [file pone.0260627.s002.doc]

# S2 APPENDIX

**Body weight: individual and mean values**

| **Strain** | **Route of Infection** | **Mouse No.** | **BW (g) Day 0** | **BW (g) Day 1 (26h Post Infection)** | **% BW Loss (end vs start of infection) at 26 hours** |
| --- | --- | --- | --- | --- | --- |
| ***A. baumannii* ACC001** | IN | 1 | 30.70 | / | / |
|  |  | 2 | 29.90 | / | / |
|  |  | 3 | 32.40 | / | / |
|  |  | 4 | 29.50 | / | / |
|  |  | 5 | 33.20 | / | / |
|  |  | 6 | 31.80 | 28.40 | -10.69 |
|  |  | 7 | 29.40 | 24.80 | -15.65 |
|  |  | 8 | 29.00 | 28.90 | -0.34 |
|  |  | 9 | 30.20 | 29.60 | -1.99 |
|  |  | 10 | 32.20 | 30.70 | -4.66 |
|  |  | **AVERAGE** | **30.83** | **28.48** | **-6.67** |
|  |  | **SD** | **1.47** | **2.23** | **6.38** |
|  | IT | 1 | 28.90 | / | / |
|  |  | 2 | 28.80 | / | / |
|  |  | 3 | 30.80 | / | / |
|  |  | 4 | 29.00 | / | / |
|  |  | 5 | 28.90 | / | / |
|  |  | 6 | 29.20 | 24.70 | -15.41 |
|  |  | 7 | 31.50 | 26.60 | -15.56 |
|  |  | 8 | 28.40 | 24.10 | -15.14 |
|  |  | 9 | 27.30 | 22.70 | -16.85 |
|  |  | 10 | 25.50 | 21.20 | -16.86 |
|  |  | **AVERAGE** | **28.83** | **23.86** | **-15.96** |
|  |  | **SD** | **1.66** | **2.04** | **0.83** |
|  | OP | 1 | 28.40 | / | / |
|  |  | 2 | 28.40 | / | / |
|  |  | 3 | 29.50 | / | / |
|  |  | 4 | 30.50 | / | / |
|  |  | 5 | 29.80 | / | / |
|  |  | 6 | 28.50 | 24.47 | -14.14 |
|  |  | 7 | 30.60 | 26.22 | -14.31 |
|  |  | 8 | 30.90 | 25.5 | -17.48 |
|  |  | 9 | 31.00 | 26.56 | -14.32 |
|  |  | 10 | 29.70 | 25.3 | -14.81 |
|  |  | **AVERAGE** | **29.73** | **25.61** | **-15.01** |
|  |  | **SD** | **1.02** | **0.82** | **1.40** |

| **Strain** | **Route of Infection** | **Mouse No.** | **BW (g) Day 0** | **BW (g) Day 1 (26h Post Infection)** | **% BW Loss (end vs start of infection) at 26 hours** |
| --- | --- | --- | --- | --- | --- |
| ***A. baumannii* ACC002** | **IN** | 1 | 31.40 | / | / |
|  |  | 2 | 27.00 | / | / |
|  |  | 3 | 28.10 | / | / |
|  |  | 4 | 28.90 | / | / |
|  |  | 5 | 33.30 | / | / |
|  |  | 6 | 31.40 | 32.00 | 1.91 |
|  |  | 7 | 30.80 | 31.30 | 1.62 |
|  |  | 8 | 30.90 | 32.20 | 4.21 |
|  |  | 9 | 30.70 | 28.90 | -5.86 |
|  |  | 10 | 28.60 | 25.60 | -10.49 |
|  |  | **AVERAGE** | **30.11** | **30.00** | **-1.72** |
|  |  | **SD** | **1.90** | **2.79** | **6.20** |
|  | **IT** | 1 | 30.20 | / | / |
|  |  | 2 | 29.30 | / | / |
|  |  | 3 | 27.80 | / | / |
|  |  | 4 | 30.80 | / | / |
|  |  | 5 | 30.60 | / | / |
|  |  | 6 | 30.09 | 26.80 | -10.93 |
|  |  | 7 | 26.10 | 22.80 | -12.64 |
|  |  | 8 | 33.50 | 27.30 | -18.51 |
|  |  | 9 | 29.30 | 25.70 | -12.29 |
|  |  | 10 | 31.10 | 25.70 | -17.36 |
|  |  | **AVERAGE** | **29.88** | **25.66** | **-14.35** |
|  |  | **SD** | **1.98** | **1.74** | **3.36** |
|  | **OP** | 1 | 28.20 | / | / |
|  |  | 2 | 29.40 | / | / |
|  |  | 3 | 26.70 | / | / |
|  |  | 4 | 29.70 | / | / |
|  |  | 5 | 29.80 | / | / |
|  |  | 6 | 28.00 | 23.5 | -16.07 |
|  |  | 7 | 30.70 | 26.4 | -14.01 |
|  |  | 8 | 27.20 | 23 | -15.44 |
|  |  | 9 | 28.10 | 23.1 | -17.79 |
|  |  | 10 | 29.70 | 25.3 | -14.81 |
|  |  | **AVERAGE** | **28.75** | **24.26** | **-15.63** |
|  |  | **SD** | **1.29** | **1.51** | **1.43** |

**Clinical scores**

| ***A. baumannii* ACC001_IN** | | | | | |
| --- | --- | --- | --- | --- | --- |
| **TIME (h): 26** |  |  |  |  |  |
| **Mouse No.** | **6** | **7** | **8** | **9** | **10** |
| **Body weight** | 1 | 2 | 0 | 0 | 0 |
| **Appearance** | 2 | 2 | 1 | 1 | 2 |
| **Motor activity** | 1 | 1 | 0 | 0 | 0 |
| **Posture** | 1 | 1 | 1 | 1 | 1 |
| **Respiration rate** | 1 | 1 | 0 | 0 | 1 |
| **Eyes** | 0 | 0 | 0 | 0 | 0 |
| **TOTAL SCORING** | **6** | **7** | **2** | **2** | **4** |
|  |  |  |  |  |  |
| ***A. baumannii* ACC001_IT** | | | | | |
| **TIME (h): 26** |  |  |  |  |  |
| **Mouse No.** | **6** | **7** | **8** | **9** | **10** |
| **Body weight** | 2 | 2 | 2 | 3 | 3 |
| **Appearance** | 2 | 2 | 2 | 2 | 2 |
| **Motor activity** | 1 | 1 | 1 | 1 | 1 |
| **Posture** | 1 | 1 | 1 | 1 | 1 |
| **Respiration rate** | 1 | 1 | 2 | 1 | 2 |
| **Eyes** | 0 | 0 | 0 | 2 | 2 |
| **TOTAL SCORING** | **7** | **7** | **8** | **10** | **11** |
|  |  |  |  |  |  |
| ***A. baumannii* ACC001_OP** | | | | | |
| **TIME (h): 26** |  |  |  |  |  |
| **Mouse No.** | **6** | **7** | **8** | **9** | **10** |
| **Body weight** | 2 | 2 | 3 | 2 | 2 |
| **Appearance** | 2 | 2 | 2 | 2 | 2 |
| **Motor activity** | 1 | 1 | 1 | 1 | 1 |
| **Posture** | 1 | 1 | 1 | 1 | 1 |
| **Respiration rate** | 1 | 1 | 2 | 1 | 1 |
| **Eyes** | 0 | 0 | 2 | 0 | 0 |
| **TOTAL SCORING** | **7** | **7** | **11** | **7** | **7** |

| ***A. baumannii* ACC002_IN** | | | | | |
| --- | --- | --- | --- | --- | --- |
| **TIME (h): 26** |  |  |  |  |  |
| **Mouse No.** | **6** | **7** | **8** | **9** | **10** |
| **Body weight** | 0 | 0 | 0 | 0 | 1 |
| **Appearance** | 1 | 1 | 1 | 2 | 2 |
| **Motor activity** | 0 | 0 | 0 | 0 | 1 |
| **Posture** | 0 | 0 | 0 | 1 | 1 |
| **Respiration rate** | 0 | 0 | 0 | 1 | 1 |
| **Eyes** | 0 | 0 | 0 | 0 | 0 |
| **TOTAL SCORING** | **1** | **1** | **1** | **4** | **6** |
|  |  |  |  |  |  |
| ***A. baumannii* ACC002_IT** | | | | | |
| **TIME (h): 26** |  |  |  |  |  |
| **Mouse No.** | **6** | **7** | **8** | **9** | **10** |
| **Body weight** | 1 | 2 | 3 | 2 | 3 |
| **Appearance** | 2 | 2 | 2 | 2 | 2 |
| **Motor activity** | 1 | 1 | 1 | 1 | 1 |
| **Posture** | 1 | 1 | 1 | 1 | 1 |
| **Respiration rate** | 1 | 1 | 2 | 2 | 1 |
| **Eyes** | 0 | 0 | 2 | 0 | 2 |
| **TOTAL SCORING** | **6** | **7** | **11** | **8** | **10** |
|  |  |  |  |  |  |
| ***A. baumannii* ACC002_OP** | | | | | |
| **TIME (h): 26** |  |  |  |  |  |
| **Mouse No.** | **6** | **7** | **8** | **9** | **10** |
| **Body weight** | 3 | 2 | 2 | 3 | 2 |
| **Appearance** | 2 | 2 | 2 | 2 | 2 |
| **Motor activity** | 1 | 1 | 1 | 1 | 1 |
| **Posture** | 1 | 1 | 1 | 1 | 1 |
| **Respiration rate** | 1 | 1 | 1 | 1 | 1 |
| **Eyes** | 0 | 0 | 1 | 1 | 0 |
| **TOTAL SCORING** | **8** | **7** | **8** | **9** | **7** |
