# Supplementary material for: Mouse pneumonia model by Acinetobacter baumannii multidrug resistant strains: Comparison between intranasal inoculation, intratracheal instillation and oropharyngeal aspiration techniques
Source: PLoS One. 2021 Dec 2;16(12):e0260627. doi: 10.1371/journal.pone.0260627 (PMC8638993; doi:10.1371/journal.pone.0260627)
Supplement: S4 Table — Lung BALF cytokine quantification in mice infected mice by intratracheal (IT) and oropharyngeal (OP) techniques and vehicle (VEH) or tigecycline (TGC) treated. Data expressed as mean concentration (pg/mL) ±SD. Abbreviations. IFN-gamma: interferon-gamma; IL: interleukin; KC/GRO: keratinocyte chemoattractant/human growth regulated oncogene; TNF-alpha: tumor necrosis factor-alpha. (DOC) [file pone.0260627.s004.doc]

# S4 Table

**BALF cytokines quantification**

|  | **BALF Cytokines (pg/mL) (mean ± SD)** | | | |
| --- | --- | --- | --- | --- |
|  | **INTRATRACHEAL** | | **OROPHARYNGEAL** | |
|  | **VEH** | **TGC 120 mg/kg** | **VEH** | **TGC 120 mg/kg** |
| **IFN-gamma** | 18.9 ± 4.3 | 4.2 ± 3.6 | 94.7 ± 42.3 | 5.9 ± 3.1 |
| **IL-10** | 14.2 ± 2.3 | 5.9 ± 2.1 | 16.8 ± 6.1 | 6.0 ± 4.4 |
| **IL-12p70** | 674.3 ± 17.8 | 309.7 ± 143.8 | 573.1 ± 165.6 | 201.2 ± 51.9 |
| **IL-1 beta** | 289.2 ± 151.0 | 49.9 ± 24.7 | 253.8 ± 164.0 | 41.1 ± 12.2 |
| **IL-2** | 6.2 ± 0.9 | 4.7 ± 1.5 | 4.5 ± 1.6 | 3.2 ± 0.7 |
| **IL-4** | 3.2 ± 1.3 | 1.5 ± 0.3 | 2.4 ± 0.7 | 1.2 ± 0.1 |
| **IL-5** | 5.6 ± 0.8 | 2.4 ± 0.9 | 4.6 ± 1.4 | 3.7 ±3.4 |
| **IL-6** | 14803.9 ± 5946.8 | 2476.5 ± 1890.8 | 8327.3 ± 4208.2 | 1505.6 ± 349.2 |
| **KC/GRO** | 9021.2 ± 1289.5 | 6468.6 ± 1654.7 | 6553.8 ± 1897.3 | 5061.2 ± 689.1 |
| **TNF-alpha** | 10858.3 ±194.4 | 4681.2 ± 2990.7 | 9590.7 ± 2212.3 | 3753.8 ± 1269.5 |
